# Associations Between Heart Rate Recovery Dynamics With Estradiol Levels in 20 to 60 Year-Old Sedentary Women

**DOI:** 10.3389/fphys.2018.00533

**Published:** 2018-05-15

**Authors:** Thomas Beltrame, Aparecida M. Catai, Ana C. Rebelo, Nayara Y. Tamburús, Roberta S. Zuttin, Anielle C. de Medeiros Takahashi, Ester da Silva

**Affiliations:** ^1^Department of Physiotherapy, Federal University of São Carlos, São Carlos, Brazil; ^2^Institute of Computing, University of Campinas, Campinas, Brazil; ^3^Department of Morphology, Federal University of Goiás, Goiânia, Brazil; ^4^Faculty of Social Sciences and Agriculture of Itapeva, Itapeva, Brazil

**Keywords:** aging, parasympathetic, hormone, sex, fitness

## Abstract

It is hypothesized that estradiol levels, as well as aging, influence cardiac autonomic function in women. The main aim of this study was to test the correlations between heart rate recovery (HRR) dynamics, as a proxy of cardiac autonomic function, with estradiol levels and age in women. This cross-sectional study involved 44 healthy women. Heart rate (HR) data were obtained beat-by-beat during the entire experiment. Maximal incremental exercise testing (IET) on a cycle ergometer was performed followed by 6 min of recovery. During the IET recovery period, the overall HRR dynamics were evaluated by exponential data modeling (time constant “τ”) where shorter τ indicates faster HRR adjustment. Considering the cardiac autonomic complexity, HRR dynamics were also evaluated by delta (Δ) analysis considering different HR data intervals. The relationship between HRR dynamics, estradiol levels and age was tested by Pearson product-moment correlation. The overall HRR dynamics (i.e., τ) were statistically correlated with age (*r* = 0.58, *p* < 0.001) and estradiol levels (*r* = -0.37, *p* = 0.01). The Δ analysis showed that the slower overall HRR associated with aging was a consequence of slower dynamics occurring within the 45–210 s interval, indicating slower sympathetic withdrawal. In conclusion, aging effects on HRR in women seems to be correlated with a slower sympathetic withdrawal. In addition, the cardioprotective effect previously associated with estradiol seems not to influence the autonomic modulation during exercise recovery periods in women.

## Introduction

After exercise cessation, the HR decreases toward its baseline values. The speed of this decay can be used as an important metric to assess the effects of training on fitness status (Lamberts et al., 2009). Delays in HRR are associated with poorer cardiovascular health and increased mortality (Cole et al., 1999). Accordingly, there is increasing interest in HRR as a biomarker of cardiovascular health. The behavior of HRR after exercise (i.e., HRR dynamics) has been proposed as a marker of autonomic function, training status and mortality rate (Cole et al., 1999; Daanen et al., 2012; Silva et al., 2017).

The characterization of HRR dynamics is commonly done by mathematical modeling of HR during recovery periods (Neves et al., 2011). In this technique, beat-to-beat changes in HR are recorded over time and mathematical methods are used to handle the data afterwards. This reveals an immediate reduction in HR on exercise cessation followed by an exponential decrease to resting, or near resting values, depending on the intensity and duration of the preceding exercise. The HRR is mediated by vagal reactivation and sympathetic withdrawal both acting on the sinoatrial node in the heart. The initial decrease in the heart rate is usually described to vagal reactivation and the secondary exponential decline due to a combination of sympathetic reactivation and to a lesser extent vagal activation. Increased vagal modulation (Fisher et al., 2015; Hughson and Shoemaker, 2015; Sessa et al., 2018), as well as faster HRR dynamics have been associated with a decreased risk of death (Cole et al., 1999; Shetler et al., 2001; Shcheslavskaya et al., 2010; Pecanha et al., 2014). Therefore, it is expected that modifications on sympathovagal imbalance will influence HRR dynamics.

Despite the outstanding scientific advances in autonomic HR modulation assessment (Hughson and Shoemaker, 2015; McCraty and Shaffer, 2015; Perseguini et al., 2015), the mechanisms behind sympathovagal imbalance after physical exertion remains unclear. Some authors showed that HRR adjustment after dynamic exercise is influenced by hormonal status and/or aging (Cole et al., 1999; Trevizani et al., 2012). The distinctive slower HRR response in older adults after walking activities has been associated with increased sympathetic modulation and reduced vagal activity (Chen et al., 2011; Carnethon et al., 2012; Pecanha et al., 2014), demonstrating an age-associated slower HRR due to sympathovagal imbalance.

Women have a reduction of cardiovascular health after menopause (Garcia et al., 2016). Higher predisposition to develop cardiovascular diseases in women seems to be associated with lower levels of estradiol hormone (Lee et al., 1997; Kim and Menon, 2009; Reckelhoff and Maric, 2010; Khalil, 2013; Monda et al., 2017), which drastically decreases after menopause. Estrogen (indexed here by estradiol levels) seems to induce neuronal nitric oxide production (García-Durán et al., 1999) which is involved in cardiac autonomic control due to its effect on afferent-mediated baroreflex activity and vagal activity (Buch et al., 2002). Previous study (Earnest et al., 2010) reported a negative correlation between the estradiol level and sympathetic cardiac modulation. In addition, estradiol levels seem to be related to vasodilation and coronary artery disease in women (Mercuro et al., 1999).

Considering the high incidence of coronary artery disease in women (Bello and Mosca, 2004) and that HRR dynamics characterization offers a non-invasive way to look at the cardiac autonomic control, the behavior of HR after exercise cessation should be more thoroughly investigated in women with varied ages and estradiol levels. This study aimed to investigate the association between HRR dynamics with estradiol levels in women. Since aging alone could also modify HRR dynamics, we also tested the influences of aging compared to HRR dynamics of women paired by estradiol levels. We hypothesized that lower estradiol levels in women would be related to sympathovagal imbalance, evaluated by HRR dynamics characterization.

## Materials and Methods

### Study Design and Measurements

This study was carried out in accordance with the recommendations of the Human Research Guidelines, Human Research Ethics Committee from the *Universidade Metodista de Piracicaba*. The protocol was approved by the local Human Research Ethics Committee. All subjects gave written informed consent in accordance with the Declaration of Helsinki. Participants were recruited through invitation letters, advertisements at the University campus and by phone calls.

This was a cross-sectional study comprising 44 healthy women (33 ± 11 years old, 164 ± 4 cm and 60 ± 2 kg). The study inclusion criteria were: aged between 20 to 60 years old; normal level of luteinizing, follicle-stimulating, estradiol and progesterone hormones for their age; when present, regular menstrual cycle of 28 to 30 days. The study exclusion criteria were: use of hormonal contraceptive methods in the past 10 months, skeletal muscle or joint pain; use of antihypertensive, antidepressants, anti-hypoglycemic agents, or antihypertriglyceridemic drugs; hormone replacement therapy; suspected or confirmed pregnancy. Participants were also excluded if they presented any type of cardiovascular, gynecological, musculoskeletal, or neurologic disorder or dysfunction, use of any illegal drug, smoking, unexpected menstrual cycle disorders or any gynecological condition that may alter hormonal levels. Except postmenopausal women, all participants were evaluated between the 7th and 10th days after the first day of menstruation (follicular phase). To check if premenopausal women had regular menstrual cycles and ovulation, the progesterone concentration had to be above 8.98 pmol.L^-1^ on the 21st day of the menstrual cycle.

Physical activity levels were classified according to the International Physical Activity Questionnaire (IPAQ) (Craig et al., 2003) and the function capacity was classified according to the American Heart Association. Committee on Exercise (1972). Women who had not menstruated for 12 months were considered as postmenopausal women (20% of the sample, as verified a posteriori).

Beat-by-beat HR measurement was based on a 12-lead electrocardiogram (ECG) (Miniscope II, Instramed, Porto Alegre, Rio Grande do Sul, Brazil) as previously described (Silva et al., 1994). The HR data were filtered by a 5-sample moving average algorithm, second-by-second linearly interpolated and then time aligned. When appropriate, blood pressure was manually measured with a stethoscope and sphygmomanometer (Littmann, Saint Paul, MN, United States) following the Korotkoff auscultatory method. For biochemical analysis, venous blood samples from the median cubital vein were drawn after a 12-h overnight fast. An upper forearm tourniquet was applied during the sitting position and a plain vacutainer tube was used to collect 5 ml of blood. The median cubital vein area was wiped with 70% alcohol for sterilization. The serum estradiol level was determined by enzymatic colorimetric assays (Biotecnica kit, BioSystems, Barcelona, Spain).

A metabolic cart (CPX-D/BreezeSuite 6.4.1, Medical Graphics, Saint Paul, MN, United States) was used to monitor and register breath-by-breath VO_2_ signals during the exercise testing. Before each exercise testing, the metabolic cart was calibrated following the manufacturer’s specifications.

Participants came to the laboratory on two occasions. The first visit always occurred between the 7th and the 9th days after starting menstruation for premenopausal women. During this visit, participants were firstly familiarized with all study procedures. Afterwards, venous blood was drawn by a trained professional. Blood samples were immediately conditioned in a container with an adequate temperature and sent for analysis. The second visit occurred on the day after the first visit (i.e., between the 8th and the 10th days after starting menstruation) and consisted of incremental symptom-limited exercise testing (IET, further explained). Participants were always tested in the morning between 7:00 and 10:00 am to minimize circadian influences. During all the experiments, room temperature was maintained at 22°C with relative air humidity between 50 and 60%. Participants were instructed to refrain from stimulants (such as coffee or tea) and alcoholic beverages, and to have a light meal at least 24 and 2 h before testing, respectively.

### Incremental Exercise Testing (IET)

Participant’s maximal physical exertion was obtained through the IET protocol performed on a cycle ergometer with electromagnetic brakes (Corival V2, Lode BV, Groningen, Netherlands). Before IET, when participants were in the supine position, the ECG electrodes were placed following specific guidelines (Casazza, 2004). After ∼5 min in a supine position, the blood pressure and HR were measured.

The exercise protocol comprised 60-s resting (sitting on the cycle ergometer) followed by 240 s of warming-up (free wheel exercise). Afterwards, the power output was increased to 20–25 watts per minute until the participant was exhausted. The target cadence was 60 ± 5 rpm during IET and the interruption criteria were: (1) inability to maintain the target cadence and (2) respiratory exchange ratio >1.15 (Howley et al., 1995).

For safety purposes, the blood pressure was also measured every 2 min during the IET. The test was terminated if abnormal response was observed following previous guidelines (Gibbons et al., 2002). The peak VO_2-peak_ was obtained following the American Heart Association guidelines (Balady et al., 2010).

For a better HRR dynamic characterization, the muscular metabolic demand should drop to the resting level right after IET (abrupt exercise cessation). However, sudden muscle pump cessation could increase the local metabolite accumulation by the expected decrease of local blood flow (Villar and Hughson, 2013), exacerbating the peripheral metaboreflex response (Ichinose et al., 2013). Furthermore, it is important to keep the central blood pressure in a safe range to avoid fainting during recovery. Therefore, all participants performed 2 min of active cool-down (load-free exercise at 20 ± 10 rpm) followed by 4 min of resting seated on the cycle ergometer. The HRR data for subsequent analysis was relative to 6 min after IET (recovery period).

### Data Analysis

Mathematical tools for correct HRR dynamics characterization have clinical relevance and slower HRR might indicate an impaired autonomic control (Rossiter et al., 2002; Neves et al., 2011; Simões et al., 2012). We tested and compared two data modeling techniques for HRR dynamics characterization, the Δ and exponential analysis. HRR dynamics by Δ analysis can be characterized by the difference between peak HR (HR_peak_) and the 30th (or 60th) s HR value during the recovery period (Imai et al., 1994; Otsuki et al., 2007). This simple Δ method is interesting because it can analyze the HRR within different segments of the recovery period which could offer more insights into the HR autonomic modulation after maximal physical exertion. On the other hand, the exponential analysis characterizes the entire HRR response by explicit data modeling techniques (Bartels-Ferreira et al., 2014). Despite the fact that exponential modeling does not analyze the HRR in different segments, it uses the entire HRR dataset to build a model that describes the general overview of the HR modulation during the recovery period.

### Exponential Data Modeling

Exponential data modeling was performed using a specific routine developed in OriginPro 8.0 software (OriginLab, Northampton, MA, United States). This routine fitted the HRR data into an exponential model using the Levenberg-Marquardt algorithm that finds the lowest sum of the square error to select the best equation parameters (Motulsky and Ransnas, 1987; Bearden and Moffatt, 2001; Beltrame and Hughson, 2017). Although the use of multiparameter exponential equations may present smaller fitting residuals (Motulsky and Ransnas, 1987; Bell et al., 2001), too many parameters increase the model degree of freedom and more exercise repetitions may be necessary (not applicable in a real clinical environment). In addition, it is complicated to find the physiological meaning for “extra” parameters and the best fitting approach is a balance between fitting quality and physiological meaning (Hughson and Morrissey, 1983; Hughson et al., 1988; Hughson, 2009). Therefore, this study applied a single exponential equation to fit HRR data following previous studies (Bearden and Moffatt, 2001; Bartels-Ferreira et al., 2014).

**FIGURE 1:**
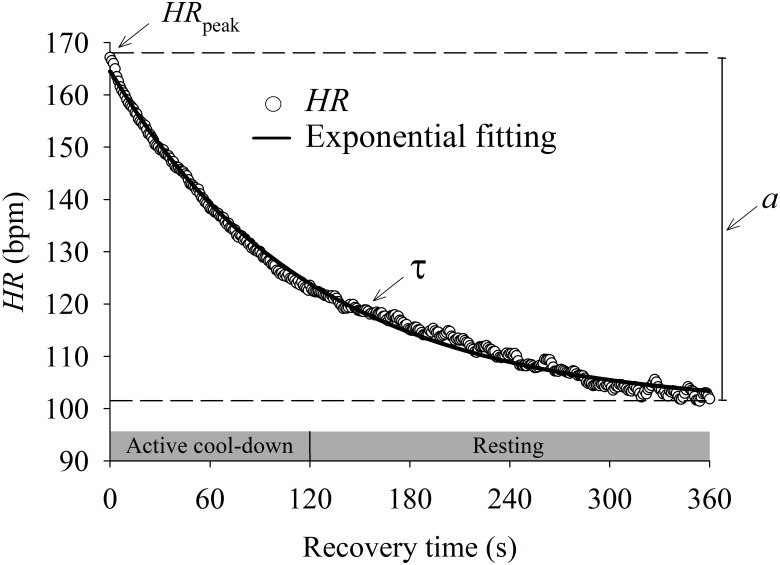
Mean (*n* = 44) heart rate (HR) recovery (HRR) response after incremental exercise protocol. Data were fitted by an exponential function (solid line) to characterize the dynamic response of HRR. The HR after the peak of exercise (HR_peak_) decreases following an exponential-like function until the steady state. The magnitude of changes from HR_peak_ to steady state HR was defined by parameter “a”. The speed of the HRR dynamics was defined by the time constant “τ.”

As described in **Figure 1**, the HRR dynamics (dependent variable) of the entire recovery period was modeled using the following exponential function (Rossiter et al., 2002):

$${HRR}_{\left(t\right)}={HR}_{peak}-a*\left(1-e^{-(t-TD)/\tau }\right)$$

where “*t*” is time (independent variable), “HR_peak_” is the peak HR at the end of the IET, “a” is the steady state HR amplitude after the end of the recovery period and “τ” is the time constant (i.e., the speed of HRR dynamics). τ is defined as the time for the HR to decrease to 63% of the final amplitude “a” after a given time delay “TD”. The parameter “TD” was included in this function because the decrease of HR might not start immediately after exercise cessation (Rossiter et al., 2002).

The data fitting quality was evaluated by residual analysis, CI_95_ (Fawkner et al., 2002; Keir et al., 2016), Pearson correlation coefficient and the statistical significance level (*p*) of the estimated parameters. As the parameter “τ” is a time constant in a negative decreasing exponential function, lower values mean faster HRR dynamics (Bearden and Moffatt, 2001; Bartels-Ferreira et al., 2014).

### Delta Analysis

The HRR data was also characterized by Δ analysis. The selected intervals of the recovery period (0–360 s, every 15 s) were subtracted from the HR_peak_. Each interval was calculated as the mean HR value measured 5 s before and after each established time point (10 s of data per interval). The higher the Δ is, the faster the HRR adjustment was because HR dropped more after a fixed period.

### Statistical Analysis

**FIGURE 2:**
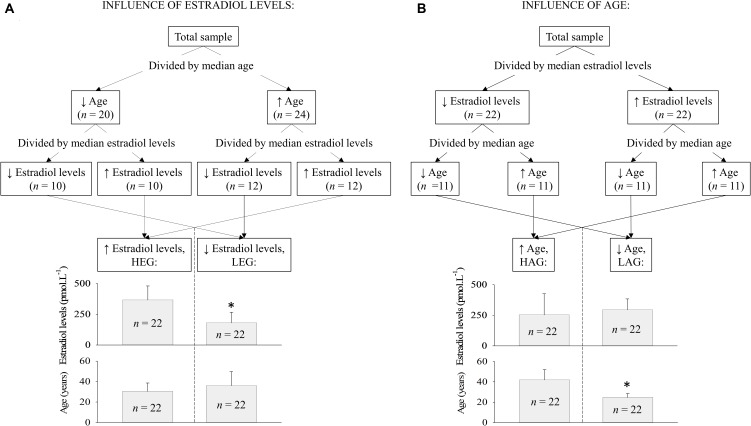
Simple median data split used to cluster the participants into groups according to estradiol levels and age. **(A)** When the variable estradiol level was isolated, there was no statistical difference in age between groups and **(B)** when the variable age was isolated, the groups presented the same estradiol level but different age. Abbreviation: HEG, higher estradiol levels group; LEG, lower estradiol levels group; HAG, higher age group; LAG, lower age group. ^∗^statistical difference by *t*-test (*p* < 0.001).

The explanatory variable estradiol levels and age were correlated (further demonstrated) indicating collinearity between the independent variables. Therefore, for statistical considerations, we decided to split the data posteriorly into different groups under the major influences of age or estradiol levels. Since age can be a confounding factor for the influence of different estradiol levels, a few data splitting techniques (including k-median, k-mean, median split, and mean split) were tested to search for the best approach to separate groups under the major influence of estradiol levels in women paired by age. Among all the tested clustering methods, the simple median split (MacCallum et al., 2002) was the best technique to statistically isolate (verified by *t*-test) the influence of estradiol levels over the influence of aging, and vice-versa. As illustrated in **Figure 2**, participants were allocated into two groups according to estradiol levels (higher estradiol levels group or HEG, *n* = 22 or lower estradiol levels group or LEG, *n* = 22) or in two groups according to age (HAG, *n* = 22 or LAG, *n* = 22).

Data were expressed as the mean ± SD. The sample size was calculated using GraphPad StatMate (version 2.00 for Windows, San Diego, CA, United States) and was based on the parameters τ and Δ obtained from a pilot study conducted with four volunteers. A two-tailed test with 80% of power and type I error of 5% was used for sample size calculation. The result indicated the need for nine participants per group (or 18 participants in total to identify age or estradiol influences). The Shapiro–Wilk test was used to assess data distribution. The Student *t*-test or Mann–Whitney Rank Sum test was used for normal and non-normal data distribution, respectively. When appropriate, multiple linear regression was used to verify age and estradiol influences simultaneously. Pearson Product Moment correlation level (*r*) (Mukaka, 2012) was used to assess correlations between two variables. The significance level established was 5% (*p* = 0.05).

## Results

The volunteers’ anthropometric characteristics and the resting cardiovascular variables are described in **Table 1**. The functional capacity of the participants is also described in **Table 1**. According to the IPAQ questionnaire, all participants were classified as “inactive” (or, “category 1”). Therefore, all participants can be considered sedentary according to IPAQ. In addition, all premenopausal women presented progesterone concentrations above 8.98 pmol.L^-1^ on the 21st day of the menstrual cycle, indicating a regular menstrual cycle and ovulation.

**Table 1:** Anthropometric characteristics, resting cardiovascular variables, peak oxygen uptake and pre and postmenopausal sample size of studied groups.

	HEG (*n* = 22)	LEG (*n* = 22)	LAG (*n* = 22)	HAG (*n* = 22)
Age (years)	30 ± 8	36 ± 13	24 ± 3	41 ± 10^b^
Estradiol (pmol⋅L^-1^)	366 ± 111	180 ± 84^a^	294 ± 87	252 ± 168
Body mass (kg)	60 ± 2	60 ± 2	59 ± 2	61 ± 2
Height (cm)	164 ± 4	164 ± 5	165 ± 3	163 ± 5
BMI [kg⋅(m^2^)^-1^]	22.38 ± 1.47	22.45 ± 1.92	21.80 ± 1.13	23.03 ± 1.95^b^
SBP (mmHg)	115 ± 4	114 ± 3	114 ± 4	115 ± 3
DBP (mmHg)	74 ± 4	74 ± 4	75 ± 4	73 ± 3
HR (bpm)	71 ± 6	68 ± 6	67 ± 5	72 ± 6^b^

		**Functional capacity^∗^**		

Low (*n*)	4	3	3	4
Fair (*n*)	14	13	14	13
Average (*n*)	3	6	5	4
Good (*n*)	1	0	0	1

		**Sample size**		

Premenopausal (*n*)	20	15	22	13
Postmenopausal (*n*)	2	7	0	9


*Data expressed as mean ± standard deviation or sample size (n). BMI, body mass index; VO_2-peak_, peak oxygen uptake; SBP, systolic blood pressure; DBP, diastolic blood pressure and HR, heart rate in resting supine position. ^*a*^statistically (p < 0.05) different between higher and lower estradiol level groups (HEG and LEG, respectively). ^*b*^statistically (p < 0.05) different between higher and lower age groups (HAG and LAG, respectively).^∗^According to the American Heart Association. Committee on Exercise (1972).*

As expected, considering the sample splitting (**Figure 2**), the estradiol levels of the HEG were statistically (*p* < 0.05) higher compared to the LEG. Likewise, age was statistically (*p* < 0.05) higher in the HAG compared to the LAG. The body mass index (BMI) and resting in supine HR were statistically (*p* < 0.05) higher in the HAG compared to the LAG. The VO_2-peak_ was statistically weakly negatively correlated with age (*r* = -0.30, *p* = 0.045), thus VO_2-peak_ seems to decrease with aging. During the experiments, the IET protocol was never terminated before the maximal physical exertion due to abnormal blood pressure or ECG responses.

Age was statistically moderately negatively correlated with estradiol levels (*r* = -0.52, *p* < 0.001), indicating that estradiol levels decrease with aging.

**FIGURE 3:**
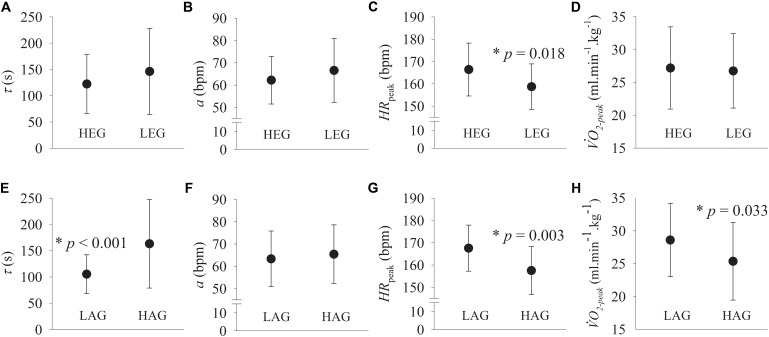
Exponential modeling parameters of heart rate recovery (HRR) data and maximal oxygen uptake (VO_2-peak_) in HEG, LEG, HAG, and LAG. The time constant (τ, in **A,E**) represents the speed of the HRR, the parameter “a” **(B,F)** is related to the magnitude of heart rate response during recovery, and peak heart rate (HR_peak_ in **C,G**) and maximal oxygen uptake (VO_2-peak_ in **D,H**) are the maximal heart rate and oxygen uptake reached during the incremental exercise, respectively. ^∗^statistically lower between groups and *p*: statistical significance level.

The comparison between groups of the exponential analysis parameters and VO_2-peak_ are described in **Figure 3**. The steady-state amplitude “a” was not statistically (*p* > 0.05) different between groups. The “HR_peak_” was statistically (*p* < 0.05) higher in the LAG and HEG compared with the HAG and LEG, respectively. The VO_2-peak_ was statistically (*p* < 0.05) higher in the LAG compared with the HAG. The time constant “τ” (speed of HRR dynamics) was statistically (*p* < 0.05) lower (i.e., faster) in the LAG compared to the HAG. The parameter “TD” (HEG: 5.13 ± 12.59, LEG: 3.72 ± 9.95, LAG: 2.90 ± 8.86, and HAG: 5.95 ± 13.25) was not different between the groups and did not present any correlation with other variables and/or parameters.

The parameter τ was statistically moderately positively correlated with age (*r* = 0.58, *p* < 0.001) and weakly negative correlated with estradiol levels (*r* = -0.37, *p* = 0.01). Using simple linear regression analysis considering age as the independent variable, τ increases ∼3.563 s (*p* < 0.001) and estradiol levels decreases ∼6 pmol⋅L^-1^ (*p* < 0.001) per year. However, estradiol levels were not statistically (*p* = 0.514) correlated with τ when age was nested in a multiple linear model. Despite the statistical difference between groups, HR_peak_ was only correlated with age (*r* = -0.479, *p* = 0.001) and seems to decrease ∼5 bpm per decade (*p* = 0.001). There was no statistical (*p* > 0.05) correlations between the parameter “a” with age and/or estrogen levels.

**FIGURE 4:**
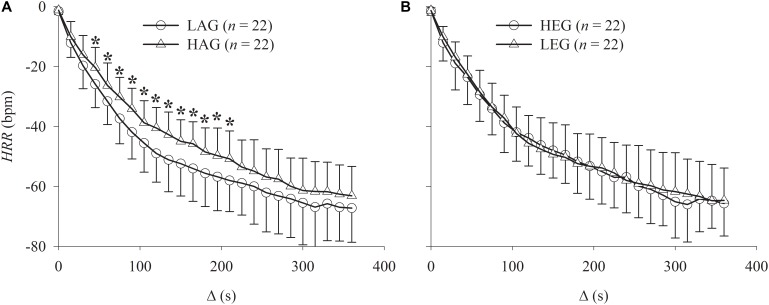
Graphs showing the comparison of the HRR relative to peak exercise (Δ analysis) after incremental exercise protocol. **(A)** Comparison between HAG and LAG. **(B)** Comparison between HEG and LEG. ^∗^Statistically different between groups.

The results of the Δ analysis at various intervals are shown in **Figure 4**. The LAG exhibited statistically (*p* < 0.05) lower HR values compared to the HAG from Δ_45_ to Δ_210_ intervals, indicating faster HRR dynamics.

**FIGURE 5:**
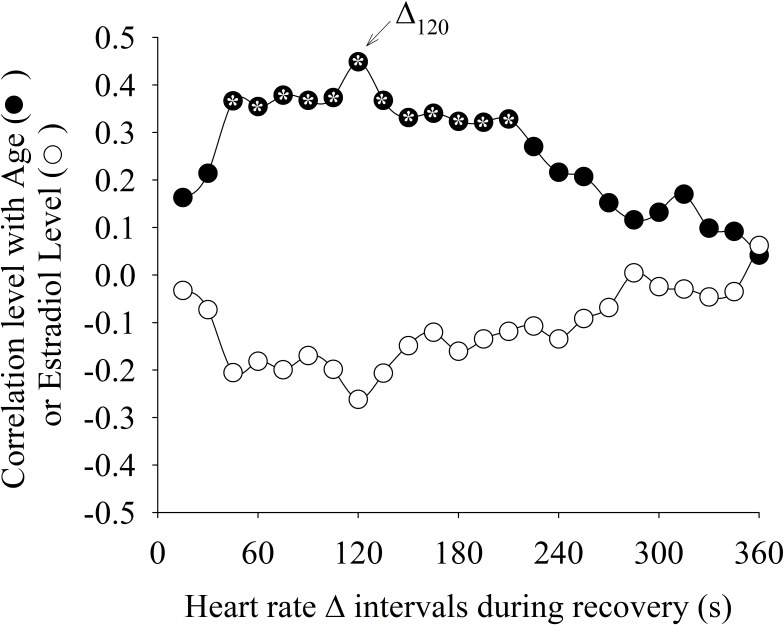
Correlation level between different heart rate delta intervals (Δ) during recovery from exercise with age and estradiol levels. The Δ interval after 120 s of recovery period (Δ_120_) presented the highest correlation with age. The “^∗^” symbol within the circle means that the correlation was statistically significant (*p* < 0.05).

**Figure 5** shows the Pearson Product Moment correlation *r* between Δ intervals with age and estradiol levels. The estradiol levels were not statistically (*p* < 0.05) correlated with any Δ interval. On the other hand, age was statistically weakly positively correlated (∼*r* = 0.37) with the intervals Δ_45_ to Δ_210_(the same intervals where statistical differences between the HAG and LAG were found). The interval Δ_120_ (arrow in **Figure 5**) presented the highest correlation with age.

**FIGURE 6:**
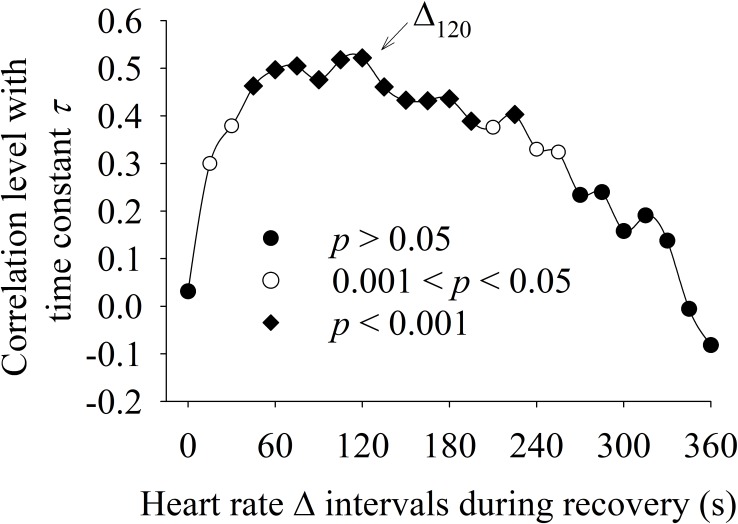
Correlation between HRR dynamics evaluated by delta analysis of different HRR intervals (Δ, *x*-axis) with the time constant “τ” obtained by exponential data analysis (*y*-axis). The Δ interval after 120 s of recovery period (Δ_120_) presented the highest correlation with “τ.”

**Figure 6** shows the correlation between the different methods used to characterize the HRR dynamics. The Δ value was statistically (*p* <0.05) positively correlated (0.3 < *r* < 0.53) with “τ” for the intervals Δ_30_ to Δ_245_. The interval Δ_120_ presented the highest correlation with “τ.”

## Discussion

The initial hypothesis that lower levels of estradiol would be related to sympathovagal imbalance in women was statistically rejected. The main findings of the present study were: (1) the HRR dynamics obtained through delta and exponential analysis were correlated only with age, not estradiol levels (2) the slower HRR associated with age seems to be linked to a slower sympathetic withdrawal occurring within the 45–210 s interval.

Although the autonomic modulation of the sinoatrial node cannot be divided into purely sympathetic or vagal control, the relative predominance of one system over the other at different time intervals must be taken into consideration (Savin et al., 1982; Imai et al., 1994). For this purpose, Δ values were calculated for specific time intervals (every 15 s) in contrast to the exponential model which includes the entire HRR response (360 s). Our results indicate that aging seems to mainly influence the HRR dynamics in the 45–210 s interval after exercise interruption. Interestingly, the time to reach 63% of the final steady-state HRR response (i.e., “τ”) was nested in this interval, explaining the higher correlation between Δ_120_ and “τ” (as demonstrated in **Figure 6**).

As described by Imai et al. (1994), who performed a pharmacological blockade, the initial reduction of the HRR is almost exclusively due to vagal reactivation that becomes supplemented by sympathetic withdrawal throughout the recovery period. According to our results, neither aging nor estrogen levels were correlated with initial HRR dynamics (before ∼30 s) suggesting no influence on early vagal reactivation.

The exponential model used in the present study proved to be sensitive to overall HRR dynamic changes and could add relevant information regarding the autonomic modulation of the heart in women (both pre and postmenopausal). To the best of our knowledge, this was the first time in the literature that slower HRR dynamics characterized by exponential analysis was associated with age in women. In addition, the simple Δ calculation was not just sensitive to changes in HRR dynamics, but also added relevant information regarding the vagal/sympathetic modulation of HR during the recovery period after peak exercise. These methods allowed us to associate the age-related slower overall HRR dynamics (higher “τ” values) with an elevated sympathetic activation within the 45–210 s interval after peak exercise.

As described in the literature (Imai et al., 1994), the time constant “τ” can be influenced by the sympathetic withdrawal and/or vagal reactivation since this method of data analysis uses the entire HRR response. However, this phenomenon has not yet been accurately described in the literature due to the difficulties of developing models that are able to distinguish between sympathetic and vagal modulation during non-steady state HR signals (Savin et al., 1982), as expected during recovery periods. Most of the studies have so far studied autonomic modulation during the resting condition (Messina et al., 2012). Unfortunately, it is during non-steady state responses (such as exercise transitions) where the physiological homeostasis is largely challenged, and non-expected/impaired responses may be detected. Therefore, methods for HRR dynamics characterization are necessary to identify modification in autonomic modulation that may not yet be presented during classic resting HR autonomic modulation assessments (Javorka et al., 2002).

The total magnitude of the HR reduction after the cessation of exercise (represented by “a”) was similar between groups and not correlated with age or estrogen level indicating that the aging process seems not to influence the magnitude of HRR changes after exercise but the speed of the HRR adjustments. As previously mentioned, higher “τ” values indicate a longer time required for the HR to recover to its baseline levels. Therefore, the higher “τ” values found in HAG might be associated to an impaired HRR adjustment associated with aging (Takahashi et al., 2002). These findings were confirmed in our results by the strong linear correlation between “τ” and age. In addition, the resting supine HR was five beats higher in the HAG compared to the LAG, which is within an expected range considering the previous literature (Fleg et al., 1990, 1994; Lakatta and Levy, 2003b). The influence of age on women HR autonomic modulation has been shown in previous studies (Cowan and Gregory, 1985; Notelovitz et al., 1986; Lee et al., 1997; Neves et al., 2007; Almeida-Santos et al., 2016). Some of these studies also identified that the decline in aerobic power after menopause was associated with age. In our study, as well as the lower HR_peak_ associated with aging, the dynamic response of the HRR was also correlated with age using different statistical approaches. Regarding the correlation between HR_peak_ and age, the lower HR_peak_ associated with aging was most likely due to a reduced beta-adrenergic response to circulating catecholamine and the lower exercise load attained at peak exercise due to reduced muscle mass (Takahashi et al., 2002; Lakatta and Levy, 2003a; Monda et al., 2008; Earnest et al., 2010).

In contrast, other authors (Correia et al., 2002; Craig et al., 2003; Mercuro et al., 2006; Bittner, 2009; Trevizani et al., 2012) stated that the impairments in aerobic power in women was an exclusive consequence of menopause, regardless of age. Our data supported these statements in part because only HR_peak_, which might be related to aerobic power, was influenced by estrogen levels in the group comparisons. However, estrogen levels were not linearly correlated with any indexes of HRR dynamics evaluated in a wide range of ages, hormone levels, and HRR data windows.

In comparison with reference values (MacNaughton et al., 1992), the estradiol levels during the follicular phase were within the normal range by age. As expected, the estrogen levels of the HEG and LEG were close to the upper and lower limit of the reference values, respectively. Therefore, the range of the estrogen levels of our participants was representative.

This study has many limitations. As demonstrated in **Table 1**, two postmenopausal women presented higher estradiol levels. This finding might be associated to the accuracy of estradiol levels measurement or to some non-diagnosed condition (such as neoplasia) that might influence the endogenous estradiol production. However, despite being classified as pre or postmenopausal woman, the aim of this study was to verify the associations between estrogen levels and HRR. Therefore, considering the context of this study, the fact that two postmenopausal women presented higher estradiol levels will not influence the interpretation of the results.

Despite the fact that the hormone levels were assessed during the follicular phase of all premenopausal women following a previous study (Casazza, 2004), estrogen may differ between the 7th and 10th days. However, considering the aim of the study and our findings, estrogen levels were not correlated to HRR, regardless of whether estrogen was different within the follicular phase.

Our study performed an active cool-down (i.e., free wheel) for 2 min after the IET which might have affected the HRR characterization. However, the energetic demand during freewheel cycling can be neglected when compared to the peak workload during IET. As described in our methods, it was important to perform a 2-min active cool-down to avoid fainting and exacerbated local metaboreflex response.

Regarding the clinical implications of the present study, the aging process alters the HRR dynamics in women, regardless of estrogen levels. In addition to previous studies (Imai et al., 1994; Cole et al., 1999; Neves et al., 2011; Silva et al., 2017), our results highlighted the clinical importance to evaluate HRR dynamics after commonly used IET. The analysis of the HRR adopting the methods proposed here may provide useful information regarding autonomic nervous control and could be easily applied in clinical setups. The Δ analysis was useful to confirm the effects of aging compared to the vagal/sympathetic modulation of the HR during exercise-to-resting transition. However, slower HRR dynamics should not be always treated as an “impaired” response. Higher HR during recovery may be necessary to buffer a diminished stroke volume (Hughson and Shoemaker, 2015) caused by lower total blood volume in older populations (Davy and Seals, 1994; Asikainen et al., 2004). Therefore, these indexes can be used as indicators of cardiovascular system adjustments in response to exercise.

## Conclusion

The present study demonstrated that HRR dynamic is slowed by aging as a consequence of modifications on the sympathetic nervous system. The cardioprotective effect previously associated with estrogen seems not to influence the autonomic modulation of the heart after peak exercise. Additionally, Δ_120_ seems to be the most reliable Δ calculation interval that can represent the entire HRR dynamic response.

## Author Contributions

TB, AC, AR, NT, RZ, AT, and EdS conceived and designed the research, performed the experiments, analyzed the data, interpreted the results of the experiments, prepared the figures, drafted the manuscript, edited and revised the manuscript, and approved the final version of the manuscript.

## Conflict of Interest Statement

The authors declare that the research was conducted in the absence of any commercial or financial relationships that could be construed as a potential conflict of interest.

## Abbreviations

τ: time constant
Δ: delta
[E]: levels of estradiol
CI_95_: 95% confidence interval band
HAG: higher age group
HEG: higher [E] group
HR: heart rate
HR_peak_: peak HR
HRR: heart rate recovery
IET: incremental exercise testing
LAG: lower age group
LEG: lower [E] group
*p*: statistical significance level
*r*: Pearson product moment correlation level
TD: time delay
VO_2_: oxygen uptake
VO_2-peak_: peak oxygen uptake
